# ASO Author Reflections: Ninety Days of Preoperative Endocrine Therapy Informs Patient and Physician Preference for Radiation Therapy

**DOI:** 10.1245/s10434-025-17996-1

**Published:** 2025-08-02

**Authors:** Lena M. Turkheimer, Trish Millard, Shayna L. Showalter

**Affiliations:** 1 Department of Surgery, University of Virginia, Charlottesville, VA USA; 2 Department of Medicine, Division of Hematology/Oncology, University of Virginia, Charlottesville, VA USA

## Past

Breast-conserving surgery (BCS) followed by radiation therapy (RT) and adjuvant endocrine therapy (AET) has long been the standard treatment for early-stage, estrogen receptor-positive (ER+) breast cancer. Randomized controlled trials, including CALGB 9343 and PRIME-II, have demonstrated that RT can be safely omitted in women aged 65 years and older who receive AET, with only a modest increase in local recurrence and no difference in overall survival.^1,2^ Despite these findings, most patients in this population still receive RT, raising concerns for overtreatment (BCS+RT+AET).^3^ RT is often recommended because patients may be non-adherent to ET, leaving those treated with BCS alone undertreated and at risk for worse oncologic outcomes.^4,5^ The decision to omit RT is made in the immediate postoperative period before patients know how well they tolerate ET. The Pre-Operative Window of Endocrine Therapy to Inform Radiation Therapy Decisions (POWER) trial was designed to evaluate whether patient tolerance of 90 days of preoperative ET (pre-ET) changes patient and physician preferences for RT.

## Present

The POWER trial demonstrated that a 90-day course of pre-ET significantly influenced both patient and physician preferences for RT. Among the 75 participants, 28% of patients changed their RT preference, and physicians changed their recommendation for 24% of the patients (*p* < 0.001 and *p* = 0.015, respectively). Importantly, patient/physician agreement regarding RT increased after the pre-ET period, likely because they could incorporate the patient’s actual experience with ET into the decision.^6^

## Future

This study validates pre-ET as a novel strategy that informs patient and physician RT preferences and facilitates individualized adjuvant treatment recommendations. The impact of pre-ET on over- and undertreatment remains to be determined. The POWER II trial (NCT06507618) is a prospective, randomized trial comparing 90 days of pre-ET with the standard of care and is currently enrolling patients at two institutions, with plans to expand. The goal of the POWER II trial is to determine whether pre-ET lowers undertreatment (BCS alone) and overtreatment (BCS+RT+ET). The results of the POWER trials have the potential to shift the treatment paradigm for older women with early-stage ER+ breast cancer.
